# DERMATOPHYTES AND RELATED KERATINOPHILIC FUNGI IN SOIL OF PARKS AND AGRICULTURAL FIELDS OF UTTAR PRADESH, INDIA

**DOI:** 10.4103/0019-5154.70700

**Published:** 2010

**Authors:** Itisha Singh, R K S Kushwaha

**Affiliations:** *From the Department of Botany, Christ Church College, Kanpur 208 001, India*; 1 *From the Department of Applied Microbiology and Biotechnology, Dr. H.S. Gour University, Sagar 470003. E-mail: kushwaharks@gmail.com*

Sir,

Indian keratinophilic fungal flora was described by Tripathi and Kushwaha[1] who describe 270 fungal species. A taxonomic review was published that dealt with 116 genera of human pathogenic keratinophilic fungi[2] and Kushwaha[3] compiled the taxonomy and biotechnological potential of *Chrysosporium* and related dermatophytes. The association of keratinophilic fungi and related dermatophytes with the soil of UP is not well documented and no data are available of the prevalence of these fungi in different parks and cultivated fields of UP. The present study was carried out to identify keratinophilic fungal flora of parks and cultivated fields of UP.

Soil samples were collected from 25 parks of Agra, Kanpur, and Lucknow and cultivated fields of 16 districts of UP, and keratinophilic fungi were isolated by using the hair-baiting technique using human hair and chicken feathers as keratinous baits. Isolated fungi were cultured and maintained on Sabouraud’s dextrose and potato dextrose agars, and also maintained as water agar cultures. The pure cultures were deposited in the Germ Plasm Centre for Keratinophilic Fungi [GPCK], Department of Botany, Christ Church College, Kanpur.

*Alternaria alternata, Aspergillus candidus, A. flavus, A. niger, Aphanoascus fulvescens, A. kiliense, Acremonium implicatum, Arthroderma tuberculatum, Aurobasidium piluliferum, Chrysosporium carmichaelii, C. georgii, C. indicum, C. keratinophilum, C. queensladicum, C. tropicum* 1, *C. tropicum* 2, *C. xerophilum, C. zonatum, C. keratinophilum, Cladosporium herbarium, Diamargaris* sp., *Geomyces pannorum, Geotrichum* sp., *Gliocladium* sp., *Gymnoascus hyalinospora, Microsporum fulvum, M. gypseum, M. vanbreuseghmii, M. mannum, Malbranchea pulchella, Mucor pusillus, Myceliophthora vellera, T. mentagrophytes, T. varrucosum,* and *Verticillium tenuipes* were isolated from 213 samples of cultivated fields of Agra, Allahabad, Etawah, Faizabad, Farrukhabad, Gonda, Gorakhpur, Kannauj, Kanpur, Lucknow, Meerut, Raibareilly, Sitapur, Unnao, Fatehpur, and Varanasi. *M. gypseum* and *C. tropicum* 1 were isolated from all the districts and yielded 51 and 44 isolates respectively; *C. keratinophilum* and *C. indicum* yielded 34 and 31 isolates. *A. alternata* was isolated from 11 districts. Among six species of *Aspergillus, A. fulvescens* was present in the soil from seven districts, *A. kiliense* from only two, and *A. niger* was the most common, showing its presence in nine districts and yielding 11 isolates. *A. implicatum* could be isolated from eight districts whereas *A. tuberculatum* was present in ten districts. *B. piluliferum* was present in only three districts whereas a maximum of ten species of *Chrysosporium* were encountered during this study. *C. herbarum, Diamrgaris* sp., *G. pannorum, Geotrichum,* and *Gliocladium* sp. could be isolated from seven, five, three, three, and five districts respectively. Ten isolates of *G. hyalinospora* were found in six districts. *M. fulvum, M. racemosum, M. mannum* were isolated from six, five, and three districts only, whereas *M. gypseum* was present in all the samples. *M. pulchella* was also common in the soil of nine districts, yielding 12 isolates. *Mucor* was restricted to only one district. *T. mentagrophytes* and *T verrucosum* were isolated in six and two districts respectively. *V. tenuipes* was restricted to five districts, representing one isolate from each. Kanpur and Lucknow yielded 65 and 62 isolates of keratinophilic fungi respectively, and Varanasi and Agra districts showed presence of 51 and 50 isolates of these fungi.

A total of 641 isolates were obtained from 125 soil samples of 25 parks of Agra, Kanpur and Lucknow and they are represented by 31 species. *A. clavatus, A. flavus, A. niger, A. parasiticus, Absidia repens, Acremonium implicatum, Aphanoasus fulvescens, Arthroderma tuberculatum, Aspergillus candidus, C. georgii, C. indicum, C. keratinophilum, C. queensladicum, C. tropicum 1, C. xerophilum, C. zonatum, Chaetomium globosum, Chrysosporium carmichaelii, Ctenomyces serratus, Curvularia lunata, Geomyces pannorum, Gliocladium sp., Gymnoascus hyalinospora, M. gypseum, M. vanbreuseghmii, M. mannum, Malbranchea pulchella, Mcrosporum fulvum, Mucor pusillus, Myceliophthora vellera, Nannizzia gypsea, T. varrucosum, T. mentagrophytes, Verticillium tenuipes* were isolated from 25 parks. Among four species of *Aspergillus*, 16 isolates of *A. niger* were found in park soil. Similarly, 16 isolates of *A. implicatum* and 31 isolates of *A. alternata* were obtained. *A. fulvescens, A. terreus,* and *A. tuberculatum* yielded ten, 20, and 22 isolates; only one *Aspergillus* species was found in parks. Eight species of *Chrysosporium* were isolated of which 48 isolates of *C. tropicum* were found. The isolate number of *C. globosum* was 53. Among dermatophytes, *M. gypseum* was the most predominant and *T. varrucosum* was represented by 29 isolates. *M. pusillus* and *M. vellera* were found in two and *C. serratus* in three parks only. Greenpark of Kanpur was found to be the richest source of keratinophilic fungi and next to this, were Gulab and Nimboo parks of Lucknow, followed by Baldev park in Agra and Surajkund park of Lucknow. CSA park, Companybagh park, and JK temple park of Kanpur yielded lower numbers of fungal isolates.

*A. flavus* first appeared on hair in three days and completely colonized hair in 12 days, which was the minimum time when compared with other fungi. *A. tuberculatum* took 14 and *A. fulvescence* took 15 days to completely colonize hair. *B. piluliferum* and *A. candidus* took the most time to colonize all the hairs, while the rest of the fungi colonized hair completely within a month. The rapid and complete colonization of hair in soil is indicative of their ability to survive on these substrates and degrade the keratin at a later stage. Filipello[4] isolated 57 species of keratinophilic fungi from public parks in Italy and found that species of *Microsporum, Aphanoascus, Chrysosporium, Malbranchea,* and *Geomyces* were the most active keratinolytic. Vidal *et al*.^[8]^ isolated keratinophilic fungi from parks of Barcelona, Spain. Gugnani *et al*.[5] obtained 23 isolates of *T. mentagrophytes,* two isolates of *M. gypseum* and several isolates of species of *Aspergillus, Penicillium, Paecilomyces, Fusarium, Chrysosporium, Acremonium, Rhizopus, Mucor, Geotrichum, Trichosoporon,* and *Rhodotourula* from different soil samples in India and Nepal. It can be concluded that the occurrence of these potentially pathogenic fungi in cultivated and park soils is of considerable importance, and their ability to perforate human hair is an evidence of their keratinolytic ability. The incidence of 50 isolates of *Microsporum* in cultivated soil and 60 isolates in parks along with large numbers of isolates of other fungi may predict the possibility of infection in human beings. Similarly, the incidence of dermatophytes and potentially pathogenic keratinophilic fungi in the soil of cultivated fields and parks, and their ability to perforate human hair is an indication of the outbreak of infections of skin and its appendages in farmers and children who remain in contact with these two habitats. This is the first report of keratinophilic genera, namely, *Absidia, Alternaria, Acremonium, Aphanoascus, Arthroderma, Aurobasidium, Cephalosporium, Cladosporium, Curvularia, Diamargaris, Geotrichum, Gliocladium, Gymnoascus, Microsporum, Malbranchea, Mucor, Myceliophthora, Nannizzia, Trichophyton,* and *Verticillium* in agricultural fields and parks of UP, India.
